# Cholesterol-lowering drugs for high-risk hypercholesterolemia patients with COVID-19 while on Paxlovid™ therapy

**DOI:** 10.2217/fvl-2022-0060

**Published:** 2022-08-02

**Authors:** Alpo Vuorio, Petri T Kovanen, Frederick Raal

**Affiliations:** ^1^Mehiläinen Airport Health Centre, Vantaa, Finland; ^2^Department of Forensic Medicine, University of Helsinki, Helsinki, 00271, Finland; ^3^Wihuri Research Institute, Helsinki, 00290, Finland; ^4^Faculty of Health Sciences, University of the Witwatersrand, Johannesburg, South Africa

**Keywords:** COVID-19, ezetimibe, familial hypercholesterolemia, interaction, Paxlovid, PCSK9 inhibitors, statins

## Abstract

Paxlovid™ is a promising antiviral oral medication for patients at a high risk of a severe form of COVID-19. Regarding COVID-19 patients who have hypercholesterolemia and are at high or very high risk for an acute atherothrombotic cardiovascular event, we are highlighting patients with heterozygous familial hypercholesterolemia as an example of severe hypercholesterolemia. Unfortunately, the concomitant use of Paxlovid and a statin, which is highly dependent on cytochrome P4507A (CYP3A) for clearance, may result in significant drug interactions. Since an abrupt withdrawal of statin use may cause serious negative rebound effects on the cardiovascular system, it is essential to continue statin treatment also during the 5-day Paxlovid treatment period. During Paxlovid treatment, simvastatin and lovastatin need to be substituted with another statin, such as pravastatin or fluvastatin, while a reduction of the dose of atorvastatin and rosuvastatin is recommended.

Paxlovid™ (PF-07321332 + ritonavir) is a promising agent which can be used to treat several viral infections, including COVID-19 caused by SARS-CoV-2 [1–3]. Recent placebo-controlled phase II/III EPIC-HR (evaluation of protease inhibition for COVID-19 in high-risk patients) studies which included 1219 non-hospitalized adult high-risk patients with SARS-CoV-2 infection showed an 89% reduction in the risk of COVID-19-related hospitalization or death from any cause when a 5-day treatment with Paxlovid was initiated within 3 days of symptom onset [4]. Currently, several clinical trials with this novel oral COVID-19 antiviral treatment candidate are ongoing, and in January 2022 the EMA recommended conditional marketing authorization for Paxlovid in high-risk patients [5]. Currently, the specified high-risk target patients with COVID-19 also include patients with coronary heart disease [6].

## COVID-19 patients with heterozygous familial hypercholesterolemia as an example of high risk for atherothrombotic cardiovascular complications

Heterozygous familial hypercholesterolemia (HeFH) is the most prevalent single-gene caused metabolic disease having a prevalence of approximately one out of 300 persons, which translates to a worldwide prevalence of at least 25 million patients with HeFH [7]. As a consequence of a two- to threefold elevation in plasma low-density lipoprotein cholesterol from birth, all HeFH patients have a very high burden of cholesterol life-years already from middle age. As a result, the majority suffer from asymptomatic coronary atherosclerosis and a significant proportion from clinically overt coronary artery disease (CAD) from the age of 40 years or even younger [8,9]. Furthermore, a population-based study found that HeFH patients with COVID-19 have a higher risk for acute myocardial infarction than matched HeFH patients without COVID-19 [10].

We chose HeFH as an example of severe hypercholesterolemia since it is the most definitive indication for an efficient statin use among hypercholesterolemic patients, and since this mandatory pharmacotherapy applies throughout the life span of each HeFH patient, in other words, from early childhood to the end of life. The treatment is initiated with high-intensity statin therapy, in most cases in combination with ezetimibe [11]. Proprotein convertase subtilisin/kexin 9 (PCSK9) inhibitors are recommended in very high-risk HeFH patients if the treatment goal is not achieved on maximally tolerated statin plus ezetimibe. HeFH is also the best example of a cardiometabolic condition in which, if left untreated or undertreated, premature atherosclerotic cardiovascular disease inevitably develops, and in which hypercholesterolemia is the sole cause of atherosclerotic cardiovascular disease, particularly in the coronary arteries, so leading to CAD. Also, statin-treated hypercholesterolemic patients without HeFH may be at strongly increased risk of an acute complication of CAD during COVID-19, notably those with a past history of an acute coronary syndrome, percutaneous coronary intervention and/or coronary artery bypass grafting.

## Statins & COVID-19

In COVID-19 patients, several studies have shown a significant improvement in outcomes among patients who are statin users, of which the majority most probably had moderate if not severe hypercholesterolemia before the introduction of statin therapy [12–14]. The results of such studies point to the importance of continuing statin therapy during SARS-CoV-2 infection not only in outpatient settings but also during hospitalization for COVID-19. Unfortunately, statin treatment is discontinued in more than half of hospitalized patients with COVID-19 [15]. Since the PCSK9 inhibitors also lower lipoprotein cholesterol concentration, the benefit observed with statins is likely to also apply to those using PCSK9 inhibitors [16]. Regarding the treatment of outpatients with COVID-19, no data are available on the prevalence of discontinuation of statin therapy. Of note, at least based on our personal experiences in outpatient care, patients often express concerns about the safety and appropriateness of taking their standard lipid-lowering medication during SARS-CoV-2 infection, and so may spontaneously stop taking their medicine.

## Paxlovid & statins

According to the information provided by Pfizer, Paxlovid is contraindicated with drugs whose clearance is highly dependent on CYP3A and for which elevated concentrations are associated with an increased risk of severe adverse reactions [17]. Differences in the CYP3A-dependent metabolism of statins and comments regarding potential interactions with Paxlovid provided by Pfizer are summarized in Table 1 [17]. In addition, interactions with statins and other cholesterol-lowering drugs are presented in Table 2 [18].

**Table 1.** Interaction Paxlovid™ with different statins according to Pfizer [17].

Statin	Clinical comments	Recommendation
Lovastatin	Highly dependent on CYP3A metabolism	Contraindicated
Simvastatin	Highly dependent on CYP3A metabolism	Contraindicated
Rosuvastatin	Less dependent on CYP3A metabolism	Use the lowest possible dose
Atorvastatin	Less dependent on CYP3A metabolism	Use the lowest possible dose
Pravastatin	Metabolism is not dependent on CYP3A metabolism	Recommended
Fluvastatin	Metabolism is not dependent on CYP3A metabolism	Recommended

**Table 2.** Interaction Paxlovid with different cholesterol lowering drugs according to University of Liverpool COVID-19 drug interactions [18].

Statin	Clinical comments	Comment
Lovastatin Simvastatin	Coadministration is contraindicated due to increased plasma concentrations; thereby, increasing the risk of myopathy including rhabdomyolysis.	Drugs that should not be coadministered
Atorvastatin	Atorvastatin is metabolized by CYP3A4 and concentrations may increase due to inhibition of CYP3A4	Potential clinically significant interaction – likely to require additional monitoring, alteration of drug dosage or timing of administration
Rosuvastatin	While rosuvastatin elimination is not dependent on CYP3A, an elevation of rosuvastatin exposure has been reported with ritonavir coadministration. The mechanism of this interaction is not clear but may be the result of transporter inhibition	Potential clinically significant interaction – likely to require additional monitoring, alteration of drug dosage or timing of administration
Pravastatin Fluvastatin Pitavastatin Evolocumab, Fenofibrate	Based on metabolism and clearance a clinically significant interaction is unlikely	
Ezetimibe	Coadministration may decrease ezetimibe concentrations	No prior dosage adjustment is recommended

## Statins, Paxlovid & heterozygous familial hypercholesterolemia

Since high-intensity statin treatment is the cornerstone of lipid-lowering therapy in HeFH, and as most adult HeFH patients are high-risk patients for cardiovascular complications of COVID-19, the above consideration of the potentially significant drug interactions and, on the other hand, safe combined use of Paxlovid and statins is crucial to note when considering Paxlovid treatment in COVID-19 patients with HeFH. The first concern is that a potential interaction between a contraindicated statin and Paxlovid poses an increased risk of myopathy, including rhabdomyolysis [17]. The second concern is that treatment with a contraindicated statin is discontinued without being adequately substituted with another statin (see Table 1). This concern derives from a bulk of evidence suggesting that abrupt statin withdrawal in patients under severe acute vascular stress is contraindicated and may worsen the outcomes [19]. Patients with COVID-19 requiring Paxlovid treatment fall into this category.

For example, a large national registry study in the US including 13871 patients receiving statins before hospital admission because of a non-ST segment elevation acute myocardial infarction showed that 35% of them discontinued their statin treatment during the first 24 h of hospitalization [20]. In this multivariate analysis, the patients who withdrew from statin therapy had a significantly elevated risk of in-hospital death compared with those who continued statin therapy. Interestingly, the patients who withdrew from statin therapy had a similar risk of hospital death to those not taking statins. The authors concluded that withdrawal from statin treatment could reflect a loss of the benefits gained during statin pretreatment and that the patients may have suffered from a rebound of inflammatory processes, thrombosis as well as from a more severe endothelial dysfunction.

The inflammatory rebound effect of statin withdrawal has been demonstrated in a Brazilian study in which patients having an acute myocardial infarction discontinued statin therapy [21]. Furthermore, in hyperlipidemic rabbits, sudden withdrawal from statin treatment induced a rebound inflammatory response and endothelial dysfunction independently of lipid changes [22]. This finding is supported by an earlier human study in which withdrawal from statin treatment led to an overshoot of small G-proteins Rho and Rac with reduced nitric oxide bioavailability and accelerated superoxide production, and ultimately resulted in increased endothelial dysfunction [23]. It has also been shown in a mouse model that withdrawal from atorvastatin treatment rapidly induces atherosclerotic plaque destabilization and rebound inflammation [24].

A very recent cohort study evaluated the association between statin discontinuation and the rate of major adverse cardiovascular events among elderly patients aged 75 years or older who had been on long-term statin treatment [25]. The study showed that the patients who discontinued statin use had a significantly higher rate of such major adverse events.

## Conclusion

Paxlovid is a promising antiviral oral medication for high-risk COVID-19 patients and has proven to decrease in-hospital mortality. Regarding high-risk HeFH patients with COVID-19, the continuation of statin therapy is essential because statins seem to have a beneficial effect on the long-term prognosis of these patients, and even more importantly, it avoids the potentially dangerous negative rebound effects caused by an acute withdrawal of the drug. Such potentially dramatic effects on the cardiovascular health of COVID-19 patients apply particularly to HeFH patients, as their dysfunctional coronary arterial endothelium is under multiple attacks during the acute phase of COVID-19 and may therefore be the root cause of coronary thrombosis and ensuing acute myocardial infarction [26]. Accordingly, it is essential to continue statin treatment, but to avoid simvastatin or lovastatin and to substitute either of them with another statin, like pravastatin or fluvastatin, during the short Paxlovid treatment period of only 5 days. In patients using atorvastatin and rosuvastatin, it is recommended to decrease the dose. Of note, there are currently no data available regarding the patient use of PCSK9 inhibitors, but this therapy appears to be safe (see Table 2), and as it is given only fortnightly or monthly, most patients taking this therapy are likely to fall into the period when a further dose is not yet required, and the effect of the previous dose is still ongoing. The decision to continue PCSK9-inhibitor therapy needs to be carried out on an individual basis, particularly since Pfizer has reported that not all the mechanisms causing harmful drug–drug interactions with Paxlovid are explained merely by effects related to CYP3A-dependent drug metabolism. For the detection of any adverse reactions related to Paxlovid–statin interactions, the outpatients should be instructed to contact their treating physician without delay. Such approach is most probably more doable than routine laboratory monitoring during the short 5-day treatment period with Paxlovid [27].

## Future perspective

Paxlovid is a promising antiviral oral medication for high-risk COVID-19 patients, and its use has proven to decrease in-hospital mortality. So far, only limited clinical experience of potential drug interactions during Paxlovid treatment is available, but this experience will grow. Increasing understanding of the potential benefits and disadvantages of the parallel use of cholesterol-lowering medications and Paxlovid is particularly important, as discontinuation of lipid-lowering medication is potentially dangerous for patients at very high risk for atherothrombotic cardiovascular events, such as acute myocardial infarction. It is to be hoped that antiviral drugs without harmful interactions with lipid-lowering medications will be available in the future.

**Executive summary**

**Background**

- Paxlovid™ is a promising antiviral oral medication for high-risk COVID-19 patients.

**COVID-19 patients with heterozygous familial hypercholesterolemia as an example of high risk for atherothrombotic cardiovascular complications**

- In patients with severe hypercholesterolemia and COVID-19, as exemplified by heterozygous familial hypercholesterolemia patients with COVID-19, the continuation of statin therapy is essential.

**Paxlovid & statins**

- During Paxlovid therapy, avoid the use of simvastatin or lovastatin. But during Paxlovid therapy, the use of pravastatin or fluvastatin is appropriate. If atorvastatin or rosuvastatin is used during Paxlovid therapy, the dose needs to be reduced.
- During Paxlovid therapy, the use of pravastatin or fluvastatin is appropriate.
- If atorvastatin or rosuvastatin is used during Paxlovid therapy, the dose needs to be reduced.

## References

[1] Couzin-Frankel J. Antiviral pills could change pandemic's course. Science 374(6569), 799–800 (2021). 34762459 10.1126/science.acx9605

[2] Mahase E. Covid-19: pfizer's paxlovid is 89% effective in patients at risk of serious illness, company reports. BMJ 375, n2713 (2021). 34750163 10.1136/bmj.n2713

[3] Wang Z, Yang L. In the age of Omicron variant: paxlovid raises new hopes of COVID-19 recovery. J. Med. Virol. 294(5), 1766–1767 (2022). 10.1002/jmv.27540 34936106

[4] Pfizer. Pfizer's novel COVID-19 oral antiviral treatment candidate reduced risk of hospitalization or death by 89% in interim analysis of Phase II/IIIEPIC-HR study (2021). https://www.pfizer.com/news/press-release/press-release-detail/pfizers-novel-covid-19-oral-antiviral-treatment-candidate

[5] European Medicines Agency. 27 January 2022. COVID-19: EMA recommends conditional marketing authorisation for Paxlovid. https://www.ema.europa.eu/en/news/covid-19-ema-recommends-conditional-marketing-authorisation-paxlovid

[6] Centers for Disease Control and Prevention. 14 October 2021. Underlying medical conditions associated with higher risk for severe COVID-19: information for healthcare providers. https://www.cdc.gov/coronavirus/2019-ncov/hcp/clinical-care/underlyingconditions.html 34009770

[7] Wilemon KA, Patel J, Aguilar-Salinas C Representatives of the Global Familial Hypercholesterolemia Community. ; Reducing the clinical and public health burden of familial hypercholesterolemia: a global call to action. JAMA Cardiol. 5(2), 217–229 (2020). 31895433 10.1001/jamacardio.2019.5173

[8] Mabuchi H, Koizumi J, Shimizu M, Takeda R. Development of coronary heart disease in familial hypercholesterolemia. Circulation 79(2), 225–232 (1989). 2914343 10.1161/01.cir.79.2.225

[9] Vuorio AF, Turtola H, Piilahti KM, Repo P, Kanninen T, Kontula K. Familial hypercholesterolemia in the Finnish North Karelia. A molecular, clinical, and genealogical study. Arterioscler. Thromb. Vasc. Biol. 17(11), 3127–3138 (1997). 9409302 10.1161/01.atv.17.11.3127

[10] Myers KD, Wilemon K, McGowan MP COVID-19 associated risks of myocardial infarction in persons with familial hypercholesterolemia with or without ASCVD. Am. J. Prev. Cardiol. 7, 100197 (2021). 34611637 10.1016/j.ajpc.2021.100197 PMC8387291

[11] Authors/Task Force Members, ESC Committee for Practice Guidelines (CPG), & ESC National Cardiac Societies. 2019 ESC/EAS guidelines for the management of dyslipidaemias: lipid modification to reduce cardiovascular risk. Atherosclerosis 290, 140–205 (2019). 31591002 10.1016/j.atherosclerosis.2019.08.014

[12] Chow R, Im J, Chiu N The protective association between statins use and adverse outcomes among COVID-19 patients: a systematic review and meta-analysis. PLOS ONE 16, e0253576 (2021). 34166458 10.1371/journal.pone.0253576 PMC8224908

[13] Talasaz AH, Sadeghipour P, Aghakouchakzadeh M Investigating lipid-modulating agents for prevention or treatment of COVID-19: JACC state-of-the-art review. J. Am. Coll. Cardiol. 78(16), 1635–1654 (2021). 34649702 10.1016/j.jacc.2021.08.021 PMC8504484

[14] Wu KS, Lin PC, Chen YS, Pan TC, Tang PL. The use of statins was associated with reduced COVID-19 mortality: a systematic review and meta-analysis. Ann. Med. 53(1), 874–884 (2021). 34096808 10.1080/07853890.2021.1933165 PMC8189130

[15] Vuorio A, Kovanen PT. Comment on: “Prior treatment with statins is associated with improved outcomes of patients with COVID-19: data from the SEMI-COVID-19 registry”. Drugs 81(9), 1125–1127 (2021). 34047956 10.1007/s40265-021-01537-7 PMC8160556

[16] Vuorio A, Kovanen PT. PCSK9 inhibitors for COVID-19: an opportunity to enhance the antiviral action of interferon in patients with hypercholesterolaemia. J. Intern. Med. 289(5), 749–751 (2021). 33222314 10.1111/joim.13210 PMC7753347

[17] Pfizer. PAXLOVIDTM (PF-07321332/ritonavir) – use with HMG CoA reductase inhibitors.

[18] University of Liverpool. COVID-19 drug interactions. https://www.covid19-druginteractions.org/checker

[19] Pineda A, Cubeddu LX. Statin rebound or withdrawal syndrome: does it exist? Curr. Atheroscler. Rep. 13(1), 23–30 (2011). 21104165 10.1007/s11883-010-0148-x

[20] Spencer FA, Fonarow GC, Frederick PD Early withdrawal of statin therapy in patients with non-ST-segment elevation myocardial infarction: national registry of myocardial infarction. Arch. Intern. Med. 164(19), 2162–2168 (2004). 15505131 10.1001/archinte.164.19.2162

[21] Sposito AC, Carvalho LS, Cintra RM Rebound inflammatory response during the acute phase of myocardial infarction after simvastatin withdrawal. Atherosclerosis 207(1), 191–194 (2009). 19464010 10.1016/j.atherosclerosis.2009.04.008

[22] Chen YX, Wang XQ, Fu Y Pivotal role of inflammation in vascular endothelial dysfunction of hyperlipidemic rabbit and effects by atorvastatin. Int. J. Cardiol. 146(2), 140–144 (2011). 19570586 10.1016/j.ijcard.2009.06.019

[23] Endres M, Laufs U. Effects of statins on endothelium and signaling mechanisms. Stroke 35(1 Suppl. 11), 2708–2711 (2004). 15375300 10.1161/01.STR.0000143319.73503.38

[24] Stasinopoulou M, Kadoglou NPE, Christodoulou E Statins' withdrawal induces atherosclerotic plaque destabilization in animal model-A “rebound” stimulation of inflammation. J. Cardiovasc. Pharmacol. Ther. 24(4), 377–386 (2019). 30905179 10.1177/1074248419838499

[25] Thompson W, Morin L, Jarbøl DE Statin discontinuation and cardiovascular events among older people in Denmark. JAMA Netw. Open 4(12), e2136802 (2021). 34854906 10.1001/jamanetworkopen.2021.36802 PMC8640890

[26] Kovanen PT, Raal F, Vuorio A. Patients with familial hypercholesterolemia and COVID-19: efficient and ongoing cholesterol lowering is paramount for the prevention of acute myocardial infarction. Am. J. Prev. Cardiol. 7, 100224 (2021). 34312613 10.1016/j.ajpc.2021.100224 PMC8289723

[27] Chaipichit N, Krska J, Pratipanawatr T, Jarernsiripornkul N. Statin adverse effects: patients' experiences and laboratory monitoring of muscle and liver injuries. Int. J. Clin. Pharm. 37(2), 355–364 (2015). 25630895 10.1007/s11096-015-0068-5

